# The roles and mechanisms of hypoxia in liver fibrosis

**DOI:** 10.1186/s12967-021-02854-x

**Published:** 2021-05-01

**Authors:** Jingyao Cai, Min Hu, Zhiyang Chen, Zeng Ling

**Affiliations:** Department of Laboratory Medicine, The Second Xiangya Hospital of Central South University, Changsha, 410011 Hunan People’s Republic of China

**Keywords:** Liver fibrosis, Hypoxia, Mechanism

## Abstract

Liver fibrosis occurs in response to any etiology of chronic liver injury. Lack of appropriate clinical intervention will lead to liver cirrhosis or hepatocellular carcinoma (HCC), seriously affecting the quality of life of patients, but the current clinical treatments of liver fibrosis have not been developed yet. Recent studies have shown that hypoxia is a key factor promoting the progression of liver fibrosis. Hypoxia can cause liver fibrosis. Liver fibrosis can, in turn, profoundly further deepen the degree of hypoxia. Therefore, exploring the role of hypoxia in liver fibrosis will help to further understand the process of liver fibrosis, and provide the theoretical basis for its diagnosis and treatment, which is of great significance to avoid further deterioration of liver diseases and protect the life and health of patients. This review highlights the recent advances in cellular and molecular mechanisms of hypoxia in developments of liver fibrosis.

## Introduction

Liver fibrosis occurs in response to any etiology of almost all chronic liver diseases, which is a critical stage in the developments of liver diseases and is mainly manifested by the deposition of excessive extracellular matrix (ECM). When the synthesis of ECM is greater than consumption, liver fibrosis will lead to cirrhosis and even develop into HCC [1]. To date, the specific mechanism of liver fibrosis has not yet been fully clarified, and effective laboratory diagnosis and treatment methods are still lacking. Therefore, an early and effective clinical intervention during the early stages of liver fibrosis can slow the development of liver cirrhosis and reduce the risk of developing HCC.

Oxygen has essential functions as a nutrient for cells to survive. In the liver, the physiologically occurring oxygen gradient is a major effector of metabolic zonation. Under pathological situations, hypoxia appears to be a major determinant for liver diseases [2]. The partial pressure of oxygen at which HCC tissue grows is lower than normal liver tissue [3]. A harsh hypoxic environment leads HCC cells to turn on hypoxia response, which subsequently leads to prosurvival reactions, elevated angiogenesis, adapted metabolic alteration, tumor invasion and metastasis.

Activation of the hypoxia-inducible factor (HIF) family of transcription factors is a critical component of the cellular response to hypoxia. Hypoxia-induced alterations in gene expression are controlled by a number of oxygen-regulated transcription factors, among which HIF-1 and nuclear factor kappa B (NF-kappaB) dependent pathways are important. Hypoxia up-regulates the expression of HIF-1α. Moreover, the increased generation of free radical oxygen species during hypoxia can further stimulate the production of reactive oxygen species (ROS) by cytosolic NADPH oxidase. The increased free radicals and hypoxia directly activate the NF-kappaB or through the mitogen-activated protein kinase pathway, increasing expressions of inflammation-related factors and chemokines. Therefore, hypoxia up-regulates the expression of HIF-1α and NF-kappaB to activate its downstream target genes, thereby stimulating the activation of hepatic stellate cells (HSCs), inducing angiogenesis, epithelial–mesenchymal transition (EMT), mediating chronic inflammation and genetic modification, which are involved in the occurrence and development of liver fibrosis. Besides, hepatic sinusoidal capillarization can induce hypoxia to aggravate liver fibrosis.

In summary, liver fibrosis is associated with hypoxia which directly stimulates the occurrence of liver fibrosis. Moreover, with the further development of liver fibrosis, the excessive deposition of ECM raises the vascular resistance in the liver. When the portal vein resistance increases beyond the body’s compensatory capacity, the portal vein blood flow rate and blood flow diminish, and the liver blood oxygen supply lessens, which in turn aggravating liver hypoxia [4]. It is proved that hypoxia is closely related to the occurrence and development of liver fibrosis. Therefore, exploring the role of hypoxia in liver fibrosis will help to understand the process of liver fibrosis and provide a theoretical basis for its diagnosis and treatment. It is of great significance to avoid further deterioration of liver diseases and protect patients’ lives and health.

## Hepatic stellate cell

Hepatic stellate cells (HSCs) are presently regarded as one of the key cell types involved in the progression of liver fibrosis and in the related pathophysiological and clinical complications [1]. Following liver tissue damage, HSCs undergo a process of activation towards a phenotype characterized by increased proliferation, motility, contractility and synthesis of extracellular matrix (ECM) components to promote the formation of liver fibrosis.

Hypoxia promotes the induction and progression of liver fibrosis by activating HSCs. α-SMA expression is a marker of fibroblast activation to a myofibroblastic phenotype, and HSCs a-SMA expression is increased in the fibrotic liver in vivo [5]. Upon hypoxia stimulation, the expression of a-SMA protein was elevated in HSC line LX-2 cells comparing to normoxic LX-2 cells [6]. Under hypoxic conditions, Copple et al. cultured LX-2 cells found that the phenotype of stellate cells increasingly changed toward the typical spindle-like shape of in vitro cultured fibroblasts [7]. Hepatic stellate cells in the activated phenotype have contractile properties, causing hepatic sinusoidal vasoconstriction, reducing blood flow into the liver, and aggravating hypoxia [8]. It is speculated that hypoxia activates HSCs, and the activated HSCs further deepen the degree of hypoxia and promote the development of liver fibrosis.

The MAPK and mTOR signaling pathways are important systems that regulate intracellular signal transduction, which are involved in the occurrence and development of liver fibrosis [9, 10]. Many investigations suggested that there existed a cross talk between mitogen-activated protein kinase (MAPK)/extracellular signal-regulated kinase (ERK) and PI3K/mTOR pathways to co-regulate the proliferation and survival of the HSCs [11]. Zhao and Wang et al. found that MAPK and mTOR signaling pathways stimulated by hypoxia is essential to HIF-1α activity, including attenuation of HIF-1α ubiquitination and promotion of HIF-1α nuclear translocation, so that HIF-1α enters the nucleus to act as transcriptional factor and regulating cell survival in hypoxia. HIF-1α then acts on a series of downstream cytokines, such as connective tissue growth factor, to activate HSC [12, 13]. Soumya et al. also found that under hypoxic conditions, the up-regulation of HIF-1α induced the activation of the Notch intracellular domain (NICD) of the transcription factor Notch, which in turn increased the transient receptor potential cation channel protein 6 (TRPC6) expression in LX-2 cells. In turn, the intracellular Ca^2+^ concentration continues to increase, and the increased Ca^2+^ activates the NFAT (activated T cell calcineurin-nuclear factor) pathway to promote the synthesis of ECM [14], which indicates that hypoxia also activates the Notch pathway to induce an increase in the intracellular Ca^2+^ concentration of HSC, thereby activating HSC. In addition, elevation of Ca^2+^ in the cytoplasm induced by hypoxic stress may activate the AMPK–mTOR and PKCtheta pathway in HSC, leading to enhanced HSC autophagy and ultimately HSC activation [15].

Peroxisome-proliferator activated receptor gamma (PPARgamma) plays a pivotal role in inhibition of hepatic stellate cell activation [16]. Because PPARgamma is a key mediator of HSC activation and phenotypic changes, keeping HSC in a static state [17, 18]. Studies have shown that hypoxia directly or indirectly inhibits the expression of PPARgamma by activating the Phosphatidylinositide 3-kinases/protein kinase B (P13K/AKT) signaling pathway or activating phosphodiesterase type 5 (PDE5), which leads to the hydrolysis of cyclic guanosine monophosphate (cGMP), thereby directly or indirectly inhibiting the expression of PPARgamma, which is ultimately beneficial to hypoxia-induced HSCs activation [19]. It is proved that hypoxia may activate HSCs by inhibiting the expression of PPARgamma.

Recent studies have explored potential roles for Extracellular vesicles (EVs) in the pathogenesis of liver fibrosis initiated by activation of HSCs. EVs derived from fat-laden hepatocytes undergoing chemical hypoxia cobalt (II) chloride promote a pro-fibrotic phenotype in hepatic stellate cells. EVs released to the extracellular matrix enhanced mRNA expression and protein of important fibrosis markers such as connective tissue growth factor (CTGF) and type I collagen in HSCs. In conclusion, EVs released by fat-laden hepatocytes mediates liver fibrosis by activating hepatic stellate cell [20]. At the same time, the exosomes released by HSCs activated by cobalt (II) chloride affect the metabolic switch of liver nonparenchymal cells via delivery of glycolysis-related proteins. These findings represent a novel mechanism that contributes to liver fibrosis and have significant implications for new diagnosis and treatment of liver diseases [21].

In addition, hyperbaric oxygen was used to treat CCl_4_-induced liver fibrosis rats and free radical antagonists were added to eliminate excessive free radicals produced by hyperbaric oxygen, which not only alleviated liver injury and delayed the progress of liver fibrosis, but also significantly decreased expression of matrix Metallo proteinase-2 (MMP-2) in HSC. It is speculated that MMP-2 promotes the formation and development of liver fibrosis, mainly through the regulation of the activation, proliferation, and migration of HSCs [22]. Based on the existing knowledge, we hypothesize that hypoxia activates HSCs through different signaling pathways, leading to the occurrence and development of liver fibrosis. However, if the degree of hypoxia is severe, it can also induce HSC apoptosis. A research suggests for the first time that nuclear GAPDH plays a pivotal role in promoting HSCs apoptosis under hypoxic conditions [23] (Table 1).

**Table 1:** Hypoxia-mediated HSCs activation is involved in the regulation of liver fibrosis by multiple mechanisms

Objects	Treatments	Targets	Mechanisms of action in HSCs	Refs
Primary HSC	Cultured in 1% oxygen and 5% CO_2_ balanced with N_2_	Ca^2+^	Trigger Ca^2+^–AMPK–mTOR and PKCh activation, which leads to enhanced HSC autophagy	[12]
HSC-T6	Paeoniflorin	RTKs	HSCs inactivation through mTOR/HIF-1α signaling pathway	[9]
	Cultured in 1% oxygen	HIF-1α	Hif-1α and MAPK co-regulate activation of HSC	[10]
	Rosiglitazone	PPARγ	sGC/cGMP/PKG and PI3K/AKT signals act on PPARγ synergistically to attenuate hypoxia-induced HSC activation	[16]
LX-2	Stimulated by CoCl_2_	TRPC6	1. Elevation of intracellular calcium which is coupled with the activation of the calcineurin-NFAT pathway which activates the synthesis of ECM 2. Activating SMAD2/3 dependent TGF-β signaling in facilitating upregulated expression of αSMA and collagen	[11]
	Cultured in 0.3% O_2_ and 5%CO_2,_ at 37 °C	HIF-1α	A positive feedback loop between HIF-1α and GAPDH, which promoting HSCs apoptosis under hypoxic conditions	[20]
	Treated with EVs from hepatocytes which were treated with fatty acids and CoCl_2_	EVs crosstalk	EVs from fat-laden hepatocytes undergoing chemical hypoxia evoke pro-fibrotic responses in LX-2 cells	[17]
	Stimulated by CoCl_2_	HIF-1α	Exosomes derived by HSCs was regulated by Hif-1. Exosomes containing glycolysis related proteins were involved in the activation and metabolic switch of HSCs and other liver nonparenchymal cells	[18]

## Hepatic sinusoidal capillarization

Capillarization of hepatic sinusoid induces hypoxia. Hepatic sinusoid is the narrowest vascular structure within the liver and is the principal site of blood flow regulation. Hepatic sinusoidal capillarization, characterized by gradually shrinking fenestrae on the surface of liver sinusoidal endothelial cells (LSECs) and forming of an organized basement membrane, is an initial pathologic change associated with liver fibrosis [24]. Capillary sinusitis is a gradual process. The decrease in the number of sinusoidal endothelial windows may be due to the premise of hepatocytes injury. Hepatic sinusoidal capillaries pass through the barrier hepatocytes and weaken the exchange of oxygen between the hepatic sinusoids and hepatocytes, resulting in hypoxia. Hypoxia aggravates the damage of hepatocytes and promotes the continuous deposition of ECM in the Dirichlet space, thus leading to liver fibrosis to the liver cirrhosis. This constitutes a vicious cycle of hepatocytes damage, which lead to hepatocytes atrophy and sinusoid collapse eventually. When the hepatic sinusoids are capillary, some cells in the hepatic sinusoids and extracellular matrix components will undergo a series of changes.

Some scholars pointed out that the activation of HSC is closely related to the formation of hepatic sinusoidal capillaries [25–27]. Activated HSCs secrete MMP-2. Generally, the increase in the expression of MMP-2 is considered the beginning of the pathological process of hepatic sinusoidal capillary formation. In addition, during chronic liver injury due to various reasons, activated HSCs secrete type I collagen, laminin and other components, which are deposited on the wall of the liver sinusoids to accelerate the formation of liver fibrosis.

In the early stage of capillary formation of the liver sinusoids, phagocytes can be activated under external pathological stimuli and transfer to the hepatic sinusoids. Under the infiltration of phagocytes, the hepatic sinusoids gradually narrow or even become blocked. At the same time, phagocytes will reduce the number of endothelial cell fenestrations and a decrease in pore size, thereby accelerating the progress of hepatic sinusoidal capillary [28].

In the process of hepatic sinusoid capillaries, the pathological changes of hepatic sinusoidal endothelial cells are also prominent. The loss of fenestration of LSECs, that is, the reduction in the number and diameter of the fenestrations, can occur in the early stage of liver fibrosis. ECM composition changes induce LSEC to loose fenestra and to form a basement membrane [29]. In liver fibrosis, the number of fenestrations decreases as interstitial collagen increases in the liver, a change that correlates with deposition of extracellular matrix in the space of Disse.

In conclusion, hepatic stellate cells, phagocytes and sinusoidal endothelial cells can work together to accelerate the capillary vascularization of hepatic sinusoids, thus weakening the oxygen exchange between hepatocytes and hepatic sinusoids, resulting in hypoxia. And then accelerate the process of liver fibrosis.

## Angiogenesis

Angiogenesis refers to the biological process of forming neovessels through budding or intussusception based on the original blood vessel structure. Intrahepatic angiogenesis is closely related to liver fibrosis. Studies have found that the number of new blood vessels in the liver is directly proportional to the degree of liver fibrosis and inversely proportional to the reversibility of liver fibrosis [4]. Hypoxia is one of the main driving forces of angiogenesis. The present study provides further evidence that hepatocellular hypoxia and angiogenesis progress together with fibrogenesis after liver injury [30].

Via tightly hypoxia-regulated induction of the transcriptional activator hypoxia inducible factor (HIF), a cascade of target genes and cell surface receptors encoded by most genes which expressed in vascular endothelial cells and increase the sensitivity of endothelial cells to angiogenic factors, as well as accelerate the process of liver fibrosis [31]. Recent studies have shown that hypoxia in liver tissue accompanying or artificially induced during the progression of liver fibrosis upregulates vascular endothelial growth factor (VEGF) in HSCs and LSECs through its effector molecule HIF-1α. The expression of VEGF gene induces the transformation of LSECs into capillary endothelium [32], resulting in a significant reduction or even disappearance of the sieve on the surface of the hepatic sinusoid, thereby weakening the exchange of oxygen between the hepatic sinusoids and hepatocytes, resulting in hypoxia [33]. Alterations in hepatic architecture may stimulate the development of liver fibrosis, increase the intrahepatic vascular resistance, and even develop into portal hypertension. However, inhibiting the expression of HIF-1α can slow down neovascularization: for example, the traditional Chinese medicine XFZY can reduce the synthesis and secretion of VEGF by inhibiting the expression of HIF-1α, thereby slowing down the angiogenesis [34]. Based on the above research results, HIF-1α plays a role in hypoxia-induced angiogenesis in liver fibrosis through its regulatory function on the VEGF gene expression. As a canonical signaling pathway, the hedgehog (Hh) pathway plays a key role in hypoxia-induced angiogenesis by regulating the expression of HIF-1α. The Hh pathway is activated in response to many types of liver injury. Activation of Hh pathway can not only lead to LSECs capillary vascularization, but also regulate HIF-1 α to activate HSCs angiogenesis, thus up regulating the expression of angiogenesis promoting genes [35, 36]. On the other hand, studies have found that Prospero Homeobox 1 (PROX1) can inhibit the expression of HIF-1α: CCl_4_-induced liver fibrosis mice, curcumin inhibits LSECs angiogenesis by regulating the Glis-PROX1-HIF-1α signaling pathway, thus slowing down the development of liver fibrosis [37]. At the same time, hypoxia up-regulates HIF-1α to induce the overexpression of Angiopoietin-1 (Ang-1), a key molecule in the regulation of vascular development. Ang-1 binds to its specific receptor Tie-2 and recruits mural cells to wrap around endothelial cells, promoting the development of liver fibrosis. It was found that Ang-1 gene was overexpressed in the liver tissue of CCl_4_-induced liver fibrosis rats and its specific receptor Tie-2 are also significantly up-regulated [38].

In addition to HIF-1α, HIF-2α also plays an important role in hypoxia-induced angiogenesis. Burkitt et al. found that knocking out the HIF-2α gene would increase the expression of soluble VEGFR1, which sequesters VEGF and prevents its activation of VEGFR2, is a negative endogenous modulator of angiogenesis [39]. It shows that angiogenesis is initiated and regulated by many factors and is an extremely complex process that is mediated by a variety of inducing factors and includes multiple steps, and more research is needed to clarify the regulatory factors in the process of angiogenesis.

It can be seen that hypoxia is closely related to the formation of new blood vessels. Although hypoxia increases the production of growth factors for liver repair and revascularization. However, pathologic angiogenesis can be inefficient due to the immaturity and permeability of neovessels and, as a result, may be unable to correct liver hypoxia [38]. With the continuous loss of oxygen, the continuous production of these mediators will eventually lead to liver fibrosis. Therefore, pathological angiogenesis and hypoxia may play a synergistic role in the repair of damaged normal liver tissue, thus promoting the development of liver fibrosis [40].

Hypoxia-mediated HSCs activation is also closely related to angiogenesis. Studies have found that many pro-angiogenic factors secreted by HSC activation and transforming growth factor-β (TGF-β), etc. can promote the differentiation of mesenchymal cells in the liver into pericytes [41], which provides necessary conditions for the maturation of neovascularization [42]. In addition, activated HSCs also promote the accumulation of LSECs at the angiogenesis site and secrete angiogenic factors to regulate LSECs, participates in liver angiogenesis directly [43].

In summary, hypoxia is involved in the formation of new blood vessels during liver fibrosis, by regulating the expression of hundreds of genes and most genes encoding cell surface receptors and their scavengers in vascular endothelial cells and hepatic stellate cells (Table 2).

**Table 2:** Summary of the main genes and most genes encoding cell surface receptors on hypoxia-mediated angiogenesis modifications in the regulation of liver fibrosis

Angiogenic factors	Actions during angiogenesis	Role in angiogenesis in LF	Refs
VEGF	Promotes endothelial cell survival and homeostasis Promotes endothelial cell detachment from the basement membrane VEGF and Notch co-operate in an integrated intercellular feedback that functions as a “branching pattern generator”	Alterations in hepatic architecture may stimulate the development of liver fibrosis, increase the intrahepatic vascular resistance, and even develop into portal hypertension	[32, 33]
ANG1 and Tie-2	ANG1, produced by mural cells, activates its endothelial receptor Tie-2 ANG1 stabilizes vessels, promotes pericyte adhesion, and makes them leak resistant by tightening endothelial junctions	Autocrine ANG1 promotes HSC/myofibroblast migration	[29]
VEGFR1, R2	Sequesters VEGF and prevents its activation of VEGFR2	A negative endogenous modulator of angiogenesis	[30]
PDGF-BB	Recruitment of pericytes	Produced by ECs/LSECs this factor stimulates HSC proliferation, differentiation, and migration, as well as transforms HSC into myofibroblasts	[28]
ET-1	It is abundant in vascular smooth muscle cells, and its main task is to promote cell proliferation and mediate vascular contraction	Promotes the HSC contraction and the secretion of cell matrix by HSC	[27]

In fact, neovascularization plays a critical role in the progressing from liver fibrosis to cirrhosis and HCC. Due to the substantial proliferation of connective tissue, the oxygen supply is insufficient. When the tumor’s oxygen supply is insufficient to meet the needs of tumor cells, it is necessary to increase the oxygen supply through neovascularization or angiogenesis [44]. The highly dense blood vessels also increase the probability of tumor metastasis [45]. The increased HIF in the hypoxic environment of HCC tissue can increase the expression of VEGF-α, thereby inducing new blood vessel formation. The new blood vessels alleviated the hypoxia of HCC tissue to some extent. However, the new blood vessels are structurally and functionally unsound, with more excellent permeability and discontinuity, they cannot fully relieve tissue hypoxia and even cause more severe problems [46]. Circulatory hypoxia, and the high permeability of these new blood vessels, will also promote the infiltration of tumor cells into blood vessels and enhance the ability of distant metastasis, thereby making tumors highly aggressive. Besides, the increase in peroxidase under hypoxic conditions may be related to HCC angiogenesis. The above research results suggest that the formation of new blood vessels in tumor tissues under hypoxic conditions and their specific mechanisms are also an important research direction of HCC.

## Chronic inflammation

Inflammation is involved in many pathological processes of liver fibrosis, such as angiogenesis. Studies have shown that inflammatory cells secrete pro-inflammatory cytokines and pro-angiogenic growth factors, finally leading to the formation of new blood vessels during liver fibrosis. For example, neutrophils, CD4 lymphocytes, CD8 lymphocytes, eosinophils participate in the formation of neovascularization by synthesizing and secreting VEGF [47]. Tumor necrosis factor (TNF) can regulate endothelial cell migration through the MAPK/ERK pathway, while prostaglandin E2, IL-1, IL-6 promote the expression of VEGF mRNA [48]. Adhesion molecules such as E-selectin can promote the infiltration of inflammatory cells, which are highly expressed in new blood vessels. At the same time, new blood vessels can maintain the chronic inflammatory state by transporting inflammatory cells to the site of inflammation and supplying nutrients to the proliferating inflamed tissue. A central event for the induction of chronic liver disease and the promotion of liver fibrosis, and likely for liver cancer, is inflammation. Hypoxia is an important microenvironment of chronic inflammation, which mediates the accumulation of ROS and inflammatory cytokines from inflammatory cells such as Kupffer cells, leukocytes, lymphocytes, natural killer cells after liver injury, strongly linked to mechanisms of initiation or progression of chronic inflammation.

### Kupffer cells (KCs)

KCs, the resident tissue macrophages of the liver, play a central role in the pathogenesis and resolution of various liver diseases. KCs play an important role in the pathogenesis of inflammatory liver diseases leading to fibrosis [49].

Under hypoxic conditions, activated NF-κB can activate KCs [50, 51]. Then, the activated KC releases oxygen free radicals. When a large amount of free oxygen radicals produced during hypoxia exceeds the immune capacity of the organism, they react with polyunsaturated fatty acids—main components of cell membranes—to induce lipid peroxidation, causing damage to the integrity of membranes and leading to cell and mitochondrial swelling and resulting in liver damage eventually [52]. In addition, KCs also exacerbate the development of liver fibrosis by releasing cytokines such as TGF-β and TNF-α. TGF-β accelerates the process of liver fibrosis by promoting ECM deposition and the secretion of tissue inhibitors. TNF-α aggravates liver damage by chemotaxis and activation of neutrophils [53]. Therefore, hypoxia stimulates KCs activation by up-regulating the expression of NF-κB is an important way to cause liver fibrosis. The latest research found that Chinese medicine can inhibit the transcriptional activity of NF-κB and the expression of NF-κB target gene chemokine CCL2, thereby inhibiting the production of pro-inflammatory factors and pro-fibrotic factors to achieve the effect of improving liver fibrosis [54, 55]. In summary, in the process of liver damage, hypoxia can up-regulate the expression of NF-κB to stimulate KCs to produce various inflammatory factors, promote liver inflammation, and accelerate the occurrence of liver fibrosis. In conclusion, regulating the activity of NF-κB can be used as the direction of treatment of liver fibrosis.

Toll-like receptor 4 (TLR-4) activation is implicated to play a key role in both inflammatory and fibrogenic pathways, acting on KCs and participating in the process of liver fibrosis. After liver injury caused by hypoxia, due to changes in intestinal mucosal permeability and increased bacterial translocation, the expression level of lipopolysaccharide (LPS) increases in the portal vein and systemic circulation [56]. TLR4 is the receptor of LPS, LPS/TLR4 signaling in HSCs is essential for development of liver fibrosis and acts via stimulating production of chemokines that recruit KCs alongside enabling unrestricted activation of HSCs by KCs-derived profibrogenic cytokine TGF-beta. KC is the most characteristic target of LPS, and TLR4 expression also exists on activated HSC [57]. This indicates that the increase in LPS caused by hypoxia can regulate the occurrence and development of liver fibrosis by acting on HSC and KC.

### Macrophages

Adipose tissue is widely distributed in many tissues, including the liver. Its main function is to store the excess energy after a meal in the form of neutral fat. When the energy demand increases, it can be broken down through lipolysis to provide the liver with metabolic substrates for the body's needs. In obesity, fat cells will become so large that the fat tissue is hypoxic, and hypoxia can lead to liver steatosis, which can progress to liver fibrosis. Without the ability to form new healthy fat cells, the continued expansion of the existing fat cells eventually leads to cell death and further adipose tissue inflammation, which contributes to the exacerbation of liver fibrosis [58]. Therefore, macrophages play important roles in adipose tissue inflammation and its consequences.

HIF-1α overexpressed under hypoxic conditions activates M1 type macrophages, and the active cytokines secreted by it can induce the transformation of preadipocytes into inflammatory preadipocytes. Inflammatory preadipocytes can synthesize a large amount of ECM components such as COL1, fibronectin and cytoadhesin C [59]. M1 type macrophages can also surround necrotic fat cells to form a characteristic crown-like structure (CLS), which is positively correlated with adipose tissue fibrosis. In CLS, dying adipocytes can induce macrophages to express macrophage-inducible C-type lectin. Macrophage-inducible C-type lectin can induce adipose tissue fibrosis by promoting the formation of CLS, activating myofibroblasts and increasing the expression of fibrosis-related genes [60], while depletion of macrophages significantly improve adipose tissue fibrosis [61]. In addition, subcutaneous adipose tissue macrophages significantly increase the expression of TGF-βunder hypoxia, and then induce the transcription of pro-fibrosis genes [62].

In summary, in the adipose tissue with chronic inflammation, there are intricate interactions between adipocytes, preadipocytes, macrophages and other cells. All kinds of inflammatory factors and proteins regulating liver fibrosis are involved in the regulation of the synthesis and secretion of ECM components, which accelerates the development of adipose tissue fibrosis in the liver.

### HSCs

Hypoxia stimulates the activation of HSCs, and studies have shown that HSCs in both the resting and active phases are involved in chronic liver inflammation [63]. HSCs are located in the gap between liver sinusoidal endothelial cells and hepatocytes. Due to its special location, at the early stages of the liver injury, HSCs in the resting phase play an important role in the recruitment of inflammatory cells, which release many factors causing activation of HSCs [64]. The latest research shows that activated HSCs secrete a variety of pro-inflammatory cytokines including macrophage inflammatory protein-2, which indicates that activated HSCs accelerate the process of liver fibrosis by participating in chronic inflammation.

## Epigenetic modification

Epigenetic modification refers to a series of regulating processes that may induce the changes in gene activity and expression without altering DNA sequence. Several classic epigenetic mechanisms have been extensively investigated, including DNA methylation, chromatin remodeling and regulation mediated by non-coding RNAs. There is increasing evidence that many non-coding RNAs are regulated by hypoxia [65]. Non-coding RNAs, as important molecules regulating gene expression at the post-transcriptional level, participate in the occurrence and development of liver fibrosis.

One of the current research hotspots of epigenetic modification is the regulation of miRNA on liver fibrosis. The individual expression of miRNAs in plasma/serum can reach a moderate accuracy for the detection of liver fibrosis because miRNA is small in serum and has a special structure and is not easily degraded. Kumar et al. found that miRNA-31 can inhibit the expression of factor-inhibiting HIF (FIH) in the process of liver fibrosis, and then activate HSCs through the TGF-β/Smad3 pathway [66]. At the same time, miRNA-122 also has the effect of promoting fibrosis. In recent animal experiments, it has been found that isocyanate B can inhibit the production of a variety of pro-fibrotic factors through the miRNA-122/HIF-1α signaling pathway, and has a significant protective effect on non-alcoholic steatohepatitis-related liver fibrosis [67]. However, miRNA-98 can alleviate the development of liver fibrosis. Its overexpression down-regulates the expression of hepatic leukemia factor (HLF). HLF then acts on HIF-1α and interacts with the TGF-β/Smad2/3 signaling pathway to inhibit HSC activation [68].

In addition to miRNAs regulating liver fibrosis, lncRNAs are also involved in the occurrence and development of liver fibrosis. Plasmacytoma variant translocation gene 1 (PVT1) is located at 8q24 on the sense strand of the chromosome, spanning a genomic interval of more than 300 kb. The expression of PVT1 was up-regulated in the liver tissues of activated HSCs in vitro as well as a mouse model of CCl_4_-induced liver fibrosis. It was found that hypoxia significantly increased the expression of PVT1 in primary mouse HSCs. PVT1 activates the Hedgehog pathway by enhancing the methylation of Patched1 (PTCH1) and down-regulating PTCH1 expression through competitively binding miR-152, which is a driver of EMT and HSC activation in liver fibrosis [69]. Further studies have found that after PVT1 competitively binds miRNA-152, it can also increase the expression of autophagy-related gene 14 (ATG14), induce HSCs autophagy and activate HSCs [70].

Therefore, the above studies revealed two new signaling networks, PVT1-miRNA-152-PTCH1 and PVT1-miRNA-152-ATG14, which regulate the process of liver fibrosis. H19 is an imprinted gene. Yang et al. found that H19 was significantly upregulated in CCl_4_-induced rat liver fibrosis and activated HSC expression in vitro, and the opposite pattern was detected for methyl CpG binding protein 2 (MeCP2) and insulin-like growth factor Type 1 receptor (IGF1R) [71]. Studies have revealed that MeCP2-mediated methylation silencing H19 leads to overexpression of IGF1R, thereby contributing to HSCs proliferation and participating in the regulation of liver fibrosis. H19 can be transcribed to produce lncRNA H19, which was upregulated by HIF-1α at the transcription level. Increased lncRNA-H19 accelerated lipid droplet metabolism by upregulating the AMPKα/LKB1 complex formation in HSCs, which could activate HSC and promote liver fibrosis [72]. At the same time, lncRNA H19 is a negative regulator of miRNA-148b-3p and can participate in the hypoxic stress of LSEC through positive regulation of NOX4 and negative regulation of eNOS/NO signaling pathways [73], while LSEC activated by hypoxia will participate in the occurrence and development of liver fibrosis. It can be seen that the mutual regulation mechanism between lncRNA–miRNA provides a new strategy for the treatment of liver fibrosis-related diseases. It is worth mentioning that the same non-coding RNA can regulate multiple target genes, and the same target gene can be regulated by multiple non-coding RNAs. Therefore, the regulatory role of non-coding RNAs in the process of liver fibrosis is extremely complex and requires more in-depth research.

Chromatin remodeling is a series of biological processes mediated by chromatin remodeling complex, which is characterized by nucleosome changes on chromatin. Studies have shown that hypoxia can induce increased expression of Chromatin Remodeling Protein 1, BRG1. Overexpressed BRG1 interacts with HIF-1α and is recruited by the TWIST (key regulator of endothelial phenotype) promoter to activate TWIST transcribed [74]. Therefore, targeting the HIF1α-BRG1-TWIST axis may yield novel therapeutic solutions to treat liver fibrosis.

**Fig. 1:**
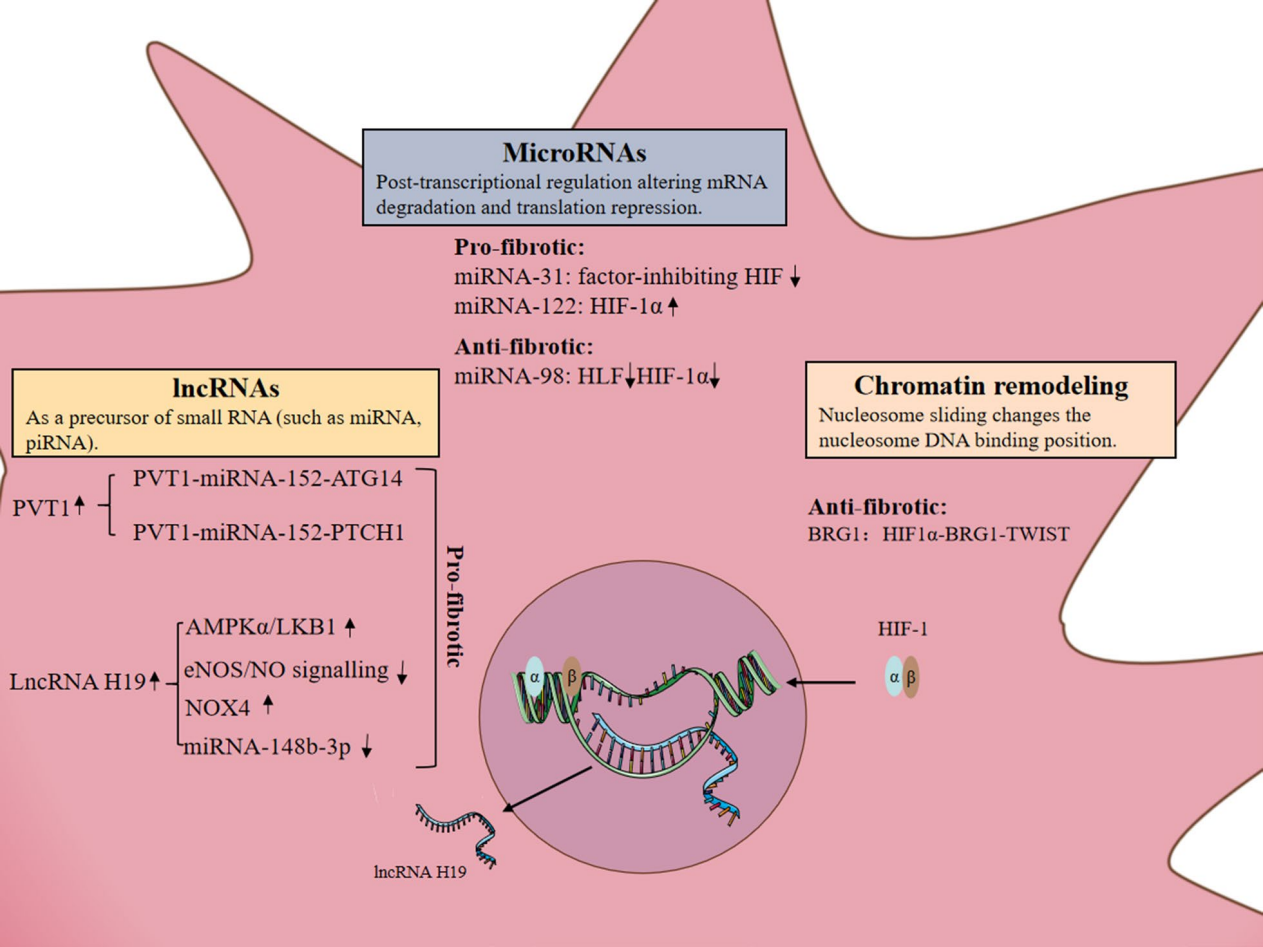
Hypoxia-mediated epigenetic modifications are involved in the regulation of liver fibrosis

In summary, epigenetic modification can participate in the effect of hypoxia on the expression of downstream target genes at the genome level, and jointly affect the pathophysiological process of liver fibrosis (Fig. 1).

## Epithelial cell–mesenchymal transition

Newly-recognized pathogenetic mechanisms of liver fibrosis point to hepatocytes undergo epithelial–mesenchymal transition (EMT) is one of the essential sources of fibroblasts. EMT refers to the biological process of epithelial cells transforming into cells with mesenchymal phenotype through specific procedures, playing a vital role in a variety of fibrotic diseases. The principal characteristics of EMT include decreased levels of cell adhesion molecules E-cadherin, the transformation of the cytokeratin cytoskeleton into the Vimentin-based cytoskeleton and the morphological characteristics of mesenchymal cells.

Hypoxia can trigger the main signal transduction pathway of the EMT process [75]: Copple et al. knocked out the HIF-1α gene in mice and found that hypoxia increases the fibroblast marker molecules such as α-SMA and vimentin in primary mouse liver cells, suggesting that hypoxia stimulates EMT of hepatocytes, thereby promoting liver fibrosis [76]. The latest research elucidates that hypoxia down-regulates the expression level of E-cadherin in HepG2 cells and up-regulates the expression level of vimentin by activating the anti-apoptotic protein Bcl-2/regulatory transcription factor Twist1 [77]. Lysyl oxidase (LOX) have recently been reported to be direct transcriptional targets of HIF-1α that induces EMT under hypoxia [78]. At the same time, the lysine residues on the collagen and fibers oxidized by LOX are conducive to the covalent cross-linking between the collagen fibers in the extracellular matrix and aggravate the process of liver fibrosis [79].

Up to now, the view of EMT as a related process in liver fibrosis is mainly based on in vitro cell culture experiments. However, a recent single-cell RNA sequencing (scRNA-seq) experiment speculates that EMT is unlikely to play a crucial role in the pathogenesis of liver fibrosis in vivo [80]. Therefore, the pathogenesis of EMT in liver fibrosis remains to be further clarified.

## Hypoxia and HIF as therapeutic targets in liver fibrosis

Because HIFs are essential regulators of profibrotic and vasoactive mediators in hypoxic hepatocytes, therapeutic inhibition of HIF-1a and HIF-2a may be beneficial in patients with liver disease to inhibit the progression of fibrosis. Such treatment may also facilitate liver regeneration, preventing the onset of liver failure and the development of HCC in patients with cirrhosis.

In recent years, it has been confirmed that liver fibrosis and a certain degree of liver cirrhosis are reversible in treatment. Some drugs promote the reversal of liver fibrosis, especially traditional Chinese medicine has unique advantages in anti-liver fibrosis, cirrhosis and HCC due to its multi-target pharmacological action. Yiguanjian Decoction has anti-angiogenic effects in CCl_4_-induced liver fibrosis mice through inhibiting HIF-1α/VEGF signaling pathway and improving the hepatic hypoxic microenvironment [81]. *Fufang Biejia Ruangan Tablets* significantly down-regulate HIF-1α mRNA, VEGF mRNA, and CTGF mRNA in liver tissues of rats. Hsu also found that green tea polyphenols by inhibiting the expression of HIF-1α, Akt signaling pathway and its downstream target gene VEGF, significantly improve intrahepatic vascular proliferation, liver fibrosis and portosystemic shunt in BDL (bile duct ligation) rats. *Astragalus membranaceus and Curcuma wenyujin* increased CD34 and reduced HIF-1α, promoted vascular normalization in tumor-derived endothelial cells of HCC [82].

Receptor tyrosine kinases (RTKs) family includes many growth factor family receptors, such as VEGF, Platelet derived growth factor (PDGF). When the ligand binds to the receptor, it activates the receptor tyrosine kinase domain and upregulates the downstream signaling system. These kinases are upregulated in liver fibrosis, cirrhosis, and HCC are considered attractive therapeutic targets. In recent years, several Tyrosine kinase inhibitors (TKIs), such as sorafenib and lenvatinib, are involved in the therapy of liver fibrosis and cirrhosis, and some other TKIs are still in clinical trials [83]. Sorafenib is the first anti-angiogenic receptor TKI, targeting VEGFR-1/2/3, PDGFR-β. Sorafenib inhibits HIF-1 α protein synthesis induced by hypoxia, lowering VEGF expression in different hepatoma cell lines and xenotransplantation mice. By blocking HIF-1α/VEGF pathway, sorafenib reduces microvessel density and inhibited tumor vascularization [84–86]. A large number of experimental studies demonstrated sorafenib’s anti-fibrotic effect. In almost all animal models of liver fibrosis, including carbon tetrachloride, BDL, dimethylnitrosamine, or diethylnitrosamine, sorafenib showed an anti-fibrotic effect [83, 87–90]. Moreover, sorafenib can reduce the wrapping of contractile HSC around LSECs, regulate the connection complex formed between LSECs, and alleviate fibrosis by inhibiting VEGF, VEGFR-2, PDGF, PDGFR-β, Tie-2 [91, 92].

Bevacizumab is a humanized monoclonal antibody against VEGF and effectively neutralizes VEGF through VEGFR-1 and VEGFR-2 receptors blocks its signal transduction and inhibits VEGF induced angiogenesis, cell proliferation, survival, permeability, nitric oxide production, migration and tissue factor production [93–95]. Bevacizumab might be a suitable agent for liver fibrosis since it alleviates liver fibrosis in vivo by neutralizing VEGF produced by hepatocytes and blocking HSCs activation [96]. In addition, Feng found bevacizumab could down-regulate the expression of α-SMA and TGF-β1, block the effect of VEGF on HSCs to reduce liver fibrosis and protect liver function [96]. It should be noted that bevacizumab treatment could raise bleeding risk and that adequate bleeding prophylaxis needs to be given [97].

Rapamycin, an immunosuppressive drug, blocked the angiogenesis of mesenteric tissue and reduced mesenteric blood flow in portal hypertensive mice, at least partly through its anti-VEGF activity and its effect on the mTOR signaling pathway [98]. A high dosage of Rapamycin can reduce hypoxia, decrease intrapulmonary shunt and improve hepatopulmonary syndrome (HPS) in cirrhotic rats by downregulating NF-κB and VEGF signaling pathway [99].

Some drugs can improve the hypoxic state of chronic liver injury by improving liver microcirculation. *Salvia miltiorrhiza* reduces platelet aggregation, accelerate microcirculation blood flow, and relieve microvascular spasm, thereby protecting the liver and promoting the repair of necrotic hepatocytes. A clinical study on the treatment of patients with liver cirrhosis using compound Danshen injection showed that the micro blood flow through the liver was significantly increased after 60 days of treatment [100]. Heparin has anticoagulant and lowers blood viscosity effects. It improves liver microcirculation. The clinical study of 34 patients with chronic HBV infection showed that after heparin/low molecular weight heparin treatment, hepatic sinus cavity blood stasis and hepatocyte swelling were significantly reduced. The pathological changes such as deposition of collagen fibers in the upper fenestration of SECs, the basement membrane and Diel cavity were improved to varying degrees [101]. It should be pointed out that liver injury is often the initiating factor of the vicious cycle of chronic liver disease and hypoxia. Therefore, early removal of the cause of liver injury and active liver protection treatment is still the key to improving the prognosis of chronic liver disease.

## Conclusion

**Fig. 2:**
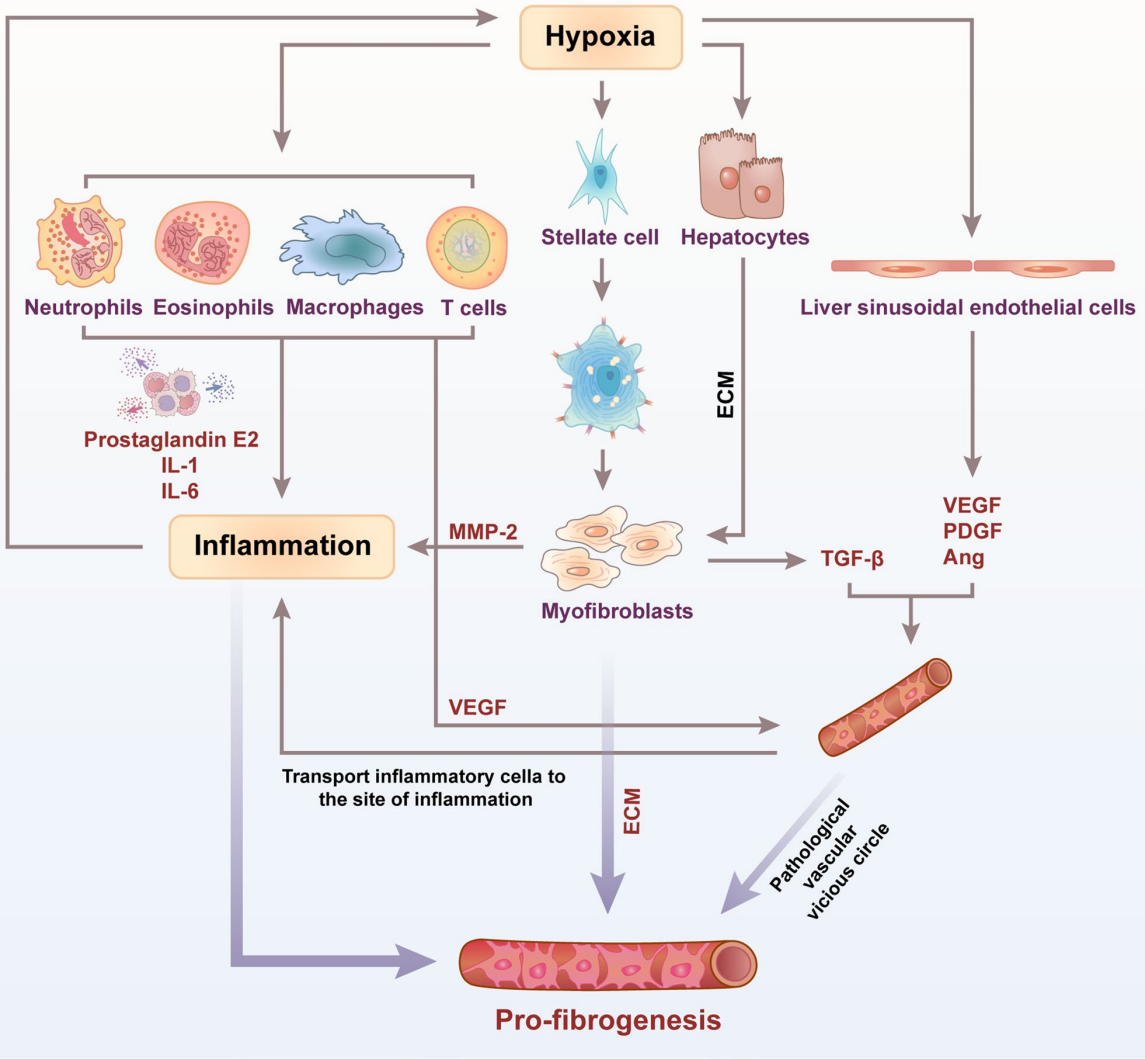
The role of hypoxia in the mechanism of liver fibrosis

As mentioned above, hypoxia participates in the occurrence and development of liver fibrosis by activating hepatic stellate cells, inducing hepatic sinusoidal capillarization and angiogenesis, mediating chronic inflammation, and epigenetic modification. As the degree of fibrosis worsens, hypoxia becomes more severe and then accelerates the process of liver fibrosis by continuously regulating related mechanisms and activating related signaling pathways. With the in-depth understanding of the mechanisms involved in HIF and its downstream targets during the occurrence and development of liver fibrosis, intervention research on HIF and its downstream targets will continue to deepen. Current research on the mechanism of HIF provides a new strategy for the treatment of liver fibrosis. However, anti-fibrosis research still needs to be verified in other liver fibrosis models and more hypoxia-inducible factor inhibitors to clarify the exact mechanism of HIF regulating liver fibrosis. Hypoxia is often not a mechanism in the occurrence and development of liver fibrosis but through multiple mechanisms that interact and promote together (Fig. 2). Due to the complex mechanisms and pathways involved between hypoxia and liver fibrosis, its exact regulatory mechanism still needs a lot of research work to further reveal to treat patients with liver fibrosis more effectively.

## Data Availability

Not applicable.

## Abbreviations

HIF: Hypoxia-inducible factor
HCC: Hepatocellular carcinoma
ECM: Excessive extracellular matrix
NF-kappaB: Nuclear factor kappa B
ROS: Reactive oxygen species
HSCs: Hepatic stellate cells
EMT: Epithelial–mesenchymal transition
MAPK: Mitogen-activated protein kinase
ERK: Extracellular signal-regulated kinase
NICD: Notch intracellular domain
TRPC6: Transient receptor potential cation channel protein 6
PPARgamma: Peroxisome-proliferator activated receptor gamma
P13K/AKT: Phosphatidylinositide 3-kinases/protein kinase B
PDE5: Phosphodiesterase type 5
cGMP: Cyclic guanosine monophosphate
EVs: Extracellular vesicles
CTGF: Connective tissue growth factor
MMP-2: Matrix Metallo proteinase-2
LSECs: Liver sinusoidal endothelial cel1s
VEGF: Vascular endothelial growth factor
PROX1: Prospero Homeobox 1
Ang-1: Angiopoietin-1
TGF-β: Transforming growth factor-β
TNF: Tumor necrosis factor
KCs: Kupffer cells
TLR-4: Toll-like receptor 4
LPS: Lipopolysaccharide
CLS: Crown-like structure
FIH: Factor-inhibiting HIF
HLF: Hepatic leukemia factor
PVT1: Plasmacytoma variant translocation gene 1
PTCH1: Patched1
ATG14: Autophagy-related gene 14
MeCP2: Methyl CpG binding protein 2
IGF1R: Insulin-like growth factor Type 1 receptor
LOX: Lysyl oxidase
scRNA-seq: Single-cell RNA sequencing
BDL: Bile duct ligation
RTK: Receptor tyrosine kinases
PDGF: Platelet derived growth factor
TKI: Tyrosine kinase inhibitor
HPS: Hepatopulmonary syndrome
